# Supplementary material for: STDP and the distribution of preferred phases in the whisker system
Source: PLoS Comput Biol. 2021 Sep 17;17(9):e1009353. doi: 10.1371/journal.pcbi.1009353 (PMC8480728; doi:10.1371/journal.pcbi.1009353)
Supplement: Attachment — Submitted filename: CoverLetter20210725.doc [file pcbi.1009353.s001.doc]

July 25, 2021

The Editor

PLOS Computational Biology

Dear Editor,

Enclosed please find a revised version of our article entitled “STDP and the distribution of preferred phases in the whisker system” by Nimrod Sherf & Maoz Shamir (yours: PCOMPBIOL-D-21-00842) that we would like to resubmit for publication in PLoS Computational Biology. We have carefully addressed the reviewers' comments. Below please find a detailed account of the modifications made to the manuscript following reviewers' suggestions. Again, we thank the reviewers for their input that clearly made our manuscript more accessible to a wider audience.

Sincerely yours,

Professor Maoz Shamir Ph.D.

**Response to reviewers' comments**

**Reviewer #1:** 
In the manuscript the authors provide a theoretical account for a puzzling experimental observation in the whisker system, i.e., the distribution of preferred phases in the cortex is not significantly sharper than that observed in the thalamus (contrary to what one would expect according to a simple "pooling" model). The non-sharpening is, according to the theory, a result of synaptic volatility induced by spike-timing dependent plasticity. The theory also makes experimental predictions that could be tested.

The results presented appear correct, as far as I can check, and novel. The assumptions underlying the modeling and its limitations are carefully discussed. The manuscript is well written and the figures informative. I found the results intriguing and certainly worth being disseminated. I have no major comment, just a curiosity: what could be the functional relevance of such a mechanism?

Response: This is an excellent question. In a broader context one could clearly inquire whether the constant remodeling of synaptic efficacies is an artefact of the `biological hardware' that the brain must overcome or whether it is a feature of an underlying principle of the CNS. At this point we cannot suggest an answer to this question. Thus, we simply raise the question and discuss possible hypotheses. See second to last paragraph of the *Discussion*.

**Reviewer #2:** 
Neurons in the VPM and in the barrel cortex are characterized by a preferred phase. The distribution of preferred phases in the two regions is non-uniform and the widths of the two distributions is comparable. In this paper, Sherf and Shamir show that this seemingly innocuous observation is in fact highly non-trivial, and discuss a possible interpretation of this result in the framework of STDP.
I find the results of this paper both interesting and novel and I strongly recommend that it would be published in PCB. However, I think that the presentation of the results is far from optimal and be substantially improved, specifically with respect to the relationship with the biological observations.
Comments:
The important observation that the widths of the distributions are comparable is NOT supported by reference 17, as claimed in line 19. I do not doubt the observation and in fact, there is evidence for it in Yu et al., Nature Neuroscience, 2016 and Kleinfeld and Deschenes, Neuron 2011, references that are cited elsewhere in the manuscript. However, I did not find it in reference 17.

Response: The references have been corrected.

Another related point that should be clarified with respect to the relation with biology is the difference between measurements that are done simultaneously and measurements done in the same animal in different days – what can we learn from the existing literature.

Response: The *Discussion* section now goes into more extensive detail on the relationship of our work to empirical works and suggests possible experimental tests of our theory for the future.

In order to explain why the fact that the two distributions are comparable is surprising, the authors discuss random or uniform pooling. Explaining why such pooling is even relevant is done only in the Discussion, even this only to some extent in the framework of random synaptic changes.

Response: We have expanded the *Introduction* section to clarify the relevance of the naïve pooling model as a comparison and highlight its discrepancies with empirical results.

I did not understand lines 244-247 and 251-252 of the Discussion section – they need to be clarified.

Response: *Lines 244-247:* We (1) expanded our explanation of the naïve model in the *Introduction* and also discuss the expected mean phase of the downstream population. (2) We added a reference to Fig 4b that shows the distribution of preferred phases without STDP. *Lines 251-252:* We now better explain how the temporal distribution of the preferred phase of a single neuron is translated into a spatial distribution over the population.

**Reviewer #3:** 
Rat and mice detect objects by rhythmically swiping there whiskers.
Thalamic neurons which track the phase of the whisking cycle encode
information about the location of the whisker. These neurons respond preferentially to
a particular phase which are narrowly distributed. Cortical neurons pool the rhythmic
excitation from several tens of thalamic neurons. Therefore, one may expect
a very narrow distribution of preferred phase of layer 4 cortical neuron narrow . However, this is not observed experimentally. The paper argues that this issue can be solved if ihalamo-cortical synapses exhibit short term dependent plasticity. To this end, the authors combine an analytical calculations and simulations.

This is is very nice piece of work. The approach is elegant. The results are convincing.
The paper is very well written and I had pleasure to read it. I have only a couple of comments.

1) Has mentioned by the authors at the end of the discussion, they neglected the recurrent interaction in layer 4. This should be emphasized much earlier.
Recurrent interactions - especially inhibition- may have a key role in broadening the
distribution of preferred phases in layer 4. This is because inhibition can de-correlate neuronal
activity. I suggest to the authors to elaborate a bit more about this alternative hypothesis.

Response: 1) We now clarify the *Introduction* that “Throughout this work we ignore the possible contribution of recurrent connection…”. 2) We now clearly state that recurrent dynamics (with an emphasis on the role of inhibition) may widen the distribution, and that this hypothesis should be further explored (in the *Discussion*). We also suggest possible experiments (thanks to your suggestion) that can assess the contribution of recurrent connectivity to the selectivity of L4I neurons to the phase of whisking.

2) In fact the paper determines the distribution of the preferred phase of the aggregate
thalamic input into cortical neurons. Optogenetic techniques combined with intracellular
recordings (i.e Lien and Scanziani, 2013, in the context of V1) makes it possible to test
the hypothesis of the paper. I think that commenting about that can be inspiring to the reader.

Response: This is an excellent point, which we now raise in the *Discussion*.
